# An essential role for the baseplate protein Gp45 in phage adsorption to *Staphylococcus aureus*

**DOI:** 10.1038/srep26455

**Published:** 2016-05-23

**Authors:** Xuehua Li, Cengiz Koç, Petra Kühner, York-Dieter Stierhof, Bernhard Krismer, Mark C. Enright, José R. Penadés, Christiane Wolz, Thilo Stehle, Christian Cambillau, Andreas Peschel, Guoqing Xia

**Affiliations:** 1 Interfaculty Institute of Microbiology and Infection Medicine, University of Tübingen, 72076 Tübingen, Germany; 2 Interfaculty Institute of Biochemistry, University of Tübingen, 72076, Tübingen, Germany; 3 Center for Plant Molecular Biology, University of Tübingen, 72076, Tübingen, Germany; 4 School of Healthcare Sciences, Manchester Metropolitan University, Chester Street, Manchester, M1 5GD, United Kingdom; 5 Institute of Infection, Immunity and Inflammation, College of Medical, Veterinary and Life Sciences, University of Glasgow, Glasgow, United Kingdom; 6 Vanderbilt University, School of Medicine, Nashville, TN 37232, USA; 7 German Center for Infection Research (DZIF), partner site Tübingen, Germany; 8 Architecture et Fonction des Macromolécules Biologiques, Centre National de la Recherche Scientifique, UMR 6098, Campus de Luminy, Case 932, 13288 Marseille Cedex 09, France; 9 Institute of Inflammation & Repair, Faculty of Medical and Human Sciences, University of Manchester, Oxford Road, Manchester, M13 9PT, United Kingdom

## Abstract

Despite the importance of phages in driving horizontal gene transfer (HGT) among pathogenic bacteria, the underlying molecular mechanisms mediating phage adsorption to *S. aureus* are still unclear. Phage ϕ11 is a siphovirus with a high transducing efficiency. Here, we show that the tail protein Gp45 localized within the ϕ11 baseplate. Phage ϕ11 was efficiently neutralized by anti-Gp45 serum, and its adsorption to host cells was inhibited by recombinant Gp45 in a dose-dependent manner. Flow cytometry analysis demonstrated that biotin-labelled Gp45 efficiently stained the wild-type *S. aureus* cell but not the double knockout mutant Δ*tarM/S*, which lacks both α- and β-O-GlcNAc residues on its wall teichoic acids (WTAs). Additionally, adsorption assays indicate that GlcNAc residues on WTAs and O-acetyl groups at the 6-position of muramic acid residues in peptidoglycan are essential components of the ϕ11 receptor. The elucidation of Gp45-involved molecular interactions not only broadens our understanding of siphovirus-mediated HGT, but also lays the groundwork for the development of sensitive affinity-based diagnostics and therapeutics for *S. aureus* infection.

Recently, there has been a renewed interest in phage-bacteria interactions because phages have not only profound influence on the biology of bacterial pathogens^1^,^2^ but also promising applications in the detection of pathogens, the biocontrol of bacterial food contamination^3^, and the treatment of bacterial infections^4^.

Phages infecting gram-positive bacteria need to adsorb and penetrate a cell envelope with a thick peptidoglycan meshwork. The mechanism of phage adsorption and genome translocation across the gram-positive cell envelope remains largely unknown for many phages, with the exception of a few dairy phages infecting *Lactobacillus, Lactococcus,* or *Streptococcus* spp. Genome comparison of several dairy phages with different host ranges enabled the identification of their receptor binding proteins (RBPs), which are essential for phage adsorption and virulence. The first RBP recognizing a gram-positive cell envelope was identified from phage Dt1 infecting *Streptococcus thermophilus*^5^. Recently, the structures of RBPs from several lactococcal phages were solved^6^. These RBPs are generally homotrimeric and are composed of three modular structures, which encompass the N-terminal shoulder domain for connection to the virion, a β-helical linker or the neck domain, and the C-terminal head domain bearing the receptor binding site for host recognition^6^.

Wall teichoic acids (WTAs) are phosphate-rich anionic glycopolymers covalently linked to the peptidoglycan in gram-positive bacteria. The two common types of WTA are either poly-1,3 glycerol-phosphate (GroP) or poly-1,5 ribitol-phosphate (RboP). The main chains of both types of WTAs can be further substituted with sugar residues and alanyl groups^7^. Previous studies on *Bacillus* phage SPP1 revealed that adsorption of this phage to its host cell initially depends on the reversible binding to WTAs, which accelerates the subsequent irreversible binding to membrane receptor YueB^8^. Interestingly, incubation of the purified SPP1 virions with recombinant YueB leads to phage DNA release *in vitro*^9^, indicating that the binding of this protein is the trigger for DNA injection.

*Staphylococcus aureus* is a gram-positive pathogen that causes not only superficial skin infections but also severe, deep tissue infections such as endocarditis, osteomyelitis, septic arthritis, and bacteraemia. It is very well known that phages or mainly siphoviruses play vital roles in the virulence, adaptation, and evolution of *S. aureus*^1^,^2^. However, it remains unclear how siphoviruses recognize *S. aureus* and what ligand-receptor interactions mediate phage adsorption to the cell surface of *S. aureus*.

Among all *S. aureus* phages, ϕ11 is probably one of the best-studied siphoviruses due to its high transducing efficiency and broad application in transducing genetic markers among *S. aureus* strains. Recently, there has been a growing interest in studying the function of ϕ11 as a helper phage mediating the horizontal gene transfer (HGT) of *S. aureus* pathogenicity islands (SaPIs)^10^. We have shown that staphylococcal siphoviruses use α-O-GlcNAc modified WTA as a receptor^11^ and that WTA structures govern phage-mediated horizontal transfer of SaPIs among major bacterial pathogens^12^. Although many structural proteins of ϕ11 have been reported^13^,^14^, its receptor binding protein (RBP) has yet to be identified. Here we report the identification and characterization of the ϕ11 RBP and the major components of its receptor in the cell wall of *S. aureus*. These data not only provide novel insight into phage-host recognition at the staphylococcal cell surface, but also establish a molecular basis to develop novel diagnostics and therapeutic treatments of *S. aureus* infection.

## Results

### Sequence analysis of the putative baseplate proteins of ϕ11

**Figure 1:**
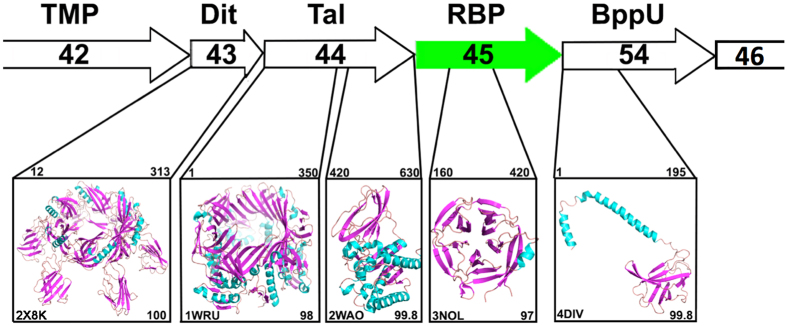
HHpred analysis of the four structural gene products following the *tmp (gp42*) of ϕ11. The genes *gp42, gp43, gp44, gp45, gp54 and gp46* are represented by arrows. The tail proteins encoded by these genes are indicated above the arrows. The structural homologues of these tail proteins are presented in the boxes beneath the corresponding genes. The PDB identifiers and ribbon structures (α-helices in blue, β-strands in violet) are shown for the structural homologues. The starting and ending amino acid residues of the regions, which could well align with these structural homologues are indicated above the boxes. The similarity probability (%) returned by HHpred is indicated to the right of the PDB identifier. The PDB entries shown here include **2** **×** **8K**, Bacillus phage SPP1 baseplate Dit protein; **1WRU**, Tail associated lysin (Tal) of bacteriophage MU; **2WAO**, carbohydrate esterase of *Clostridium thermocellum*; **3NOL**, glutaminyl cyclase of *Zymomonas mobilis*; and 4**DIV**, BppU of Lactococcus phage TP901-1.

In staphylococcal siphovirus genomes, the genes coding for tail proteins are usually located downstream of the gene of the tape measure protein (TMP) and upstream of the lysis module^2^,^15^. Among the genes localized between *tmp (gp42*) and the lysis module, *gp43, gp44, gp45* and *gp54* (Fig. 1) were previously shown to be essential for phage ϕ11 infectivity^13^,^16^. Of note, *gp54* was not initially annotated in the genome of ϕ11^17^, but it was later identified as an open reading frame localized between *gp45* and *gp46*^13^. To advance an understanding of the putative functions of the proteins encoded by these four essential genes, HHpred^18^ (Homology detection and structure prediction by HMM-HMM comparison) analysis was carried out for each protein in addition to BlastP analysis at NCBI (http://goo.gl/DE9BkO).

The HHpred analysis identified Gp43 with 100% probability as a distal tail protein (Dit) because it is similar to the Dit protein (PDB 2 × 8K) in the baseplate of the siphophage SPP1, which infects *Bacillus subtilis*^19^ (Fig. 1). The N-terminal regions of Dit proteins form a hexameric ring and are very conserved among phages^20^, although their C-terminal peripheral domains may differ considerably^21^.

BlastP search revealed that Gp44 possesses an endopeptidase domain at its N-terminus (1–350 residues) and a SGNH/GDSL hydrolase domain at its C-terminus (400–633 residues). Of note, the SGNH hydrolase represents a diverse family of lipases and esterases, but the enzyme activity of Gp44 is yet to be characterized experimentally. Further sequence analysis by HHpred revealed that the N-terminal domain of Gp44 aligns well with the tail associated lysin (Tal) of bacteriophage MU (PDB 1WRU)^22^, and its C-terminal domain exhibits striking similarity to a carbohydrate esterase (PDB 2WAO) from *Clostridium thermocellum* (Fig. 1). Tal proteins are structurally similar to Gp27, a baseplate component of the puncturing device of phage T4^23^. Notably, the gene *tal* is always localized directly downstream of the gene *dit* in siphophage genomes. In the ϕ11 tail module, *gp44* exists directly downstream of *gp43 (dit*). Hence, both sequence homology and conserved genome localization suggest that *gp44* encodes a Tal protein. Recently, it was shown that phage mutants deficient in Gp43 (Dit), or Gp44 (Tal) were defective in tails, suggesting that these two baseplate proteins are required for tail formation^16^. Furthermore, it was shown that the tail protein Gp49 possesses peptidoglycan hydrolase activity but is dispensable for ϕ11 infectivity^16^,^24^. These facts suggest that ϕ11 may have two virion-associated peptidoglycan hydrolases, Tal and Gp49, but the activity of Tal needs to be verified by further experiments.

BlastP search with Gp45 as a query returned a hit of ORF636, which shares 44% identity with Gp45 and is localized at the tail tip of phage phiSLT, a serogroup A phage of *S. aureus*. Of note, the tail protein ORF636 was characterized as an adhesion protein essential for phiSLT adsorption and infectivity^25^. HHpred analysis revealed that the central part of Gp45, covering amino acid residues 160–420, shares high similarity with 5-bladed propeller proteins (Fig. 1), for example the glutaminyl cyclase of *Zymomonas mobilis* (PDB 3NOL). The segment upstream was predicted to be α-helical by Jpred^26^, while the segment downstream was predicted to form β-strands.

Just downstream of *gp45, gp54* most likely encodes an upper baseplate protein (BppU)^27^. The N-terminus of Gp54 (amino-acids 1–195) displays high similarity to a large part of the BppU^27^, which attaches the RBP to the central baseplate core in lactococcal phage TP901-1 (Fig. 1). In TP901-1, BppU assembles as a trimer. Its N-terminus (amino-acids 1–120) is a stand-alone domain, while amino-acids 121–193 assemble as a triple α-helix bundle. This structure is followed by a trimeric all-beta domain (~100 residues), to which the N-terminus of RBP is plugged in^27^. Thus, the C-terminus of BppU and the RBP exhibit strong shape complementarity in phage TP901-1. However, the C-terminus of Gp54 possesses a domain of unknown function, which includes ~400 amino acid residues, and is much larger than that of BppU in TP901-1.

Taken together, the HHpred analyses revealed that Gp43 (Dit), Gp44 (Tal), Gp45 (ORF636-like protein), and Gp54 (BppU) very likely constitute the baseplate of ϕ11. Moreover, the central part of the ϕ11 baseplate gathering Dit, Tal, and the N-terminus of BppU, forming the dsDNA passage, is similar to that of other phages^20^, whereas the role of the tail proteins, Gp45 and Gp54, most likely located at the periphery of the baseplate is elucidated below.

### Localization of Gp45 and Gp54 at the baseplate of ϕ11

**Figure 2:**
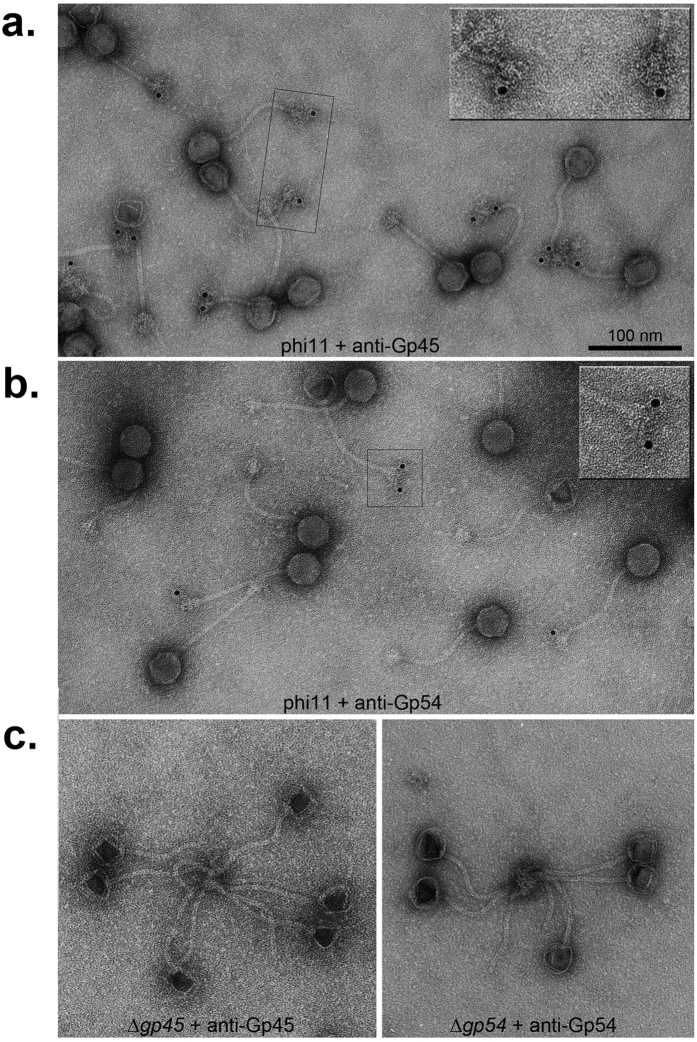
Immunogold labelling of tail proteins Gp45 and Gp54. (**a**,**b)** Transmission electron microscopy (TEM) images of negatively stained ϕ11 after immunogold labelling with anti-Gp45 serum (**a**) and anti-Gp54 serum (**b**), respectively. (**c**) TEM images of mutant phages Δ*gp45* and Δ*gp54*. (Left), mutant phage Δ*gp45* labelled with anti-Gp45 serum;(Right), mutant phage Δ*gp54* labelled with anti-Gp54 serum. Insets show enlarged views of the boxed areas.

Baseplate proteins or tail fibre proteins play critical roles in phage adsorption, the first step of phage replication cycle^6^. Previously it was shown that the two putative tail proteins Gp45 and Gp54 were essential for phage infectivity^13^. To demonstrate that both Gp54 and Gp45 are localized at the tail tip, both Gp54 and Gp45 antisera were raised and used for immunogold labelling of ϕ11. Electron micrographs of negatively stained phage samples indicate that Gp45 and Gp54 are clearly localized at the tail baseplate of ϕ11 (Fig. 2a,b), whereas immunogold labelling of mutant phages deficient in *gp45* or *gp54* resulted in negligible background labelling (Fig. 2c).

### Neutralization of ϕ11 infection with anti-Gp45 or anti-Gp54 serum

**Figure 3:**
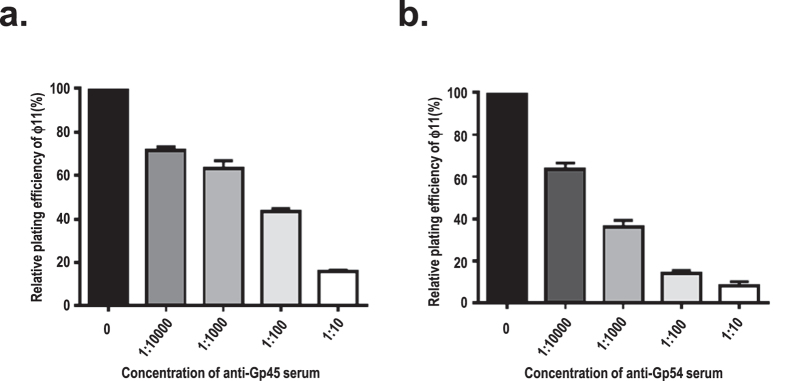
Neutralization of ϕ11 infection with rabbit anti-Gp45 or anti-Gp54 serum. Data represent means ± standard deviations (SD, n = 3). Inhibition of ϕ11 plating efficiency with anti-Gp45 (**a**) or anti-Gp54 serum (**b**) were shown respectively.

As both Gp45 and Gp54 are baseplate proteins, their roles in phage adsorption and infection were analysed. Phage ϕ11 virions were pre-incubated with increasing concentrations of antisera before plating on the host. Notably, pre-immune sera exhibited hardly any inhibitory effects on phage plating efficiency (data not shown), whereas both anti-Gp45 and anti-Gp54 serum decreases the plating efficiency of ϕ11 in a dose-dependent manner (Fig. 3a,b), which clearly suggests that these sera can specifically neutralize ϕ11 infectivity. It is most likely that masking of Gp45 or Gp54 with antiserum prevents their access to the phage receptor in the cell wall, hence blocks the phage adsorption and leads to neutralization of ϕ11.

### Gp45 binds to the cell wall with α- or β-O-GlcNAc modified WTAs

**Figure 4:**
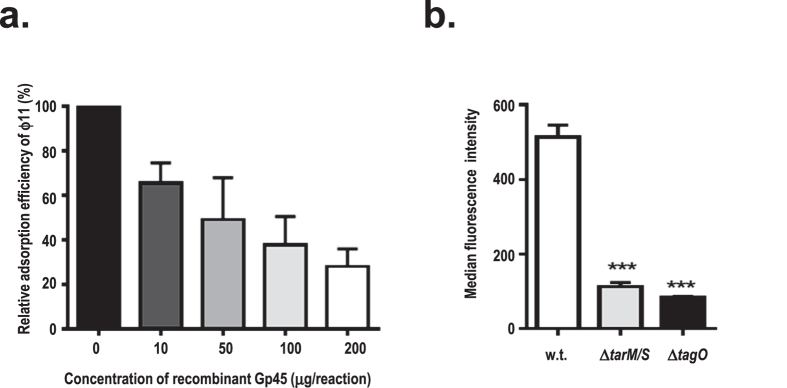
The RBP (receptor binding protein) activity of Gp45. (**a**) Dose-dependent inhibition of ϕ11 adsorption with recombinant Gp45. (**b**) Flow cytometry analysis of *S. aureus* wild-type cells and mutant cells stained with biotin-labelled Gp45. Wild-type (w.t., white bar), Δ*tarM/S* mutant (grey bar), Δ*tagO* mutant (black bar). Values are given as means ± standard deviations (SD, n = 3). Statistical significant differences calculated by one way ANOVA with Bonferroni’s post-test are indicated: ***p < 0.001.

To investigate the molecular interaction of ϕ11 with its cognate receptor on the host cell surface, recombinant Gp45 was expressed and purified (Supplementary Fig. S1). Pre-incubation of host cells with increasing concentrations of recombinant Gp45 led to dose-dependent inhibition of ϕ11 adsorption (Fig. 4a).

We recently demonstrated that *S. aureus* siphoviruses use α-O-GlcNAc modified WTAs as their adsorption receptor^11^. To examine whether Gp45 binds to WTAs, *S. aureus* wild-type strain RN4220 and mutants with altered WTAs were stained with biotin-labelled recombinant Gp45 and subsequently analysed by flow cytometry. In contrast to the well-stained wild-type *S. aureus* with glycosylated WTA, the mutants Δ*tarM/S,* which lacks α-O- and β-O-GlcNAc residues on WTA, or Δ*tagO*, which is deficient in WTA, demonstrated drastically decreased background staining (Fig. 4b). These results indicate that Gp45 binds to the cell wall with α- or β-O-GlcNAc modified WTAs. Unfortunately, recombinant Gp54 purified from *E. coli* was found to be susceptible to degradation and was therefore not suitable for flow cytometry analysis.

### The major components of the ϕ11 receptor in the cell wall of *S. aureus*

Previous studies have shown that the entire cell wall of *S. aureus* could inactivate *S. aureus* phages, while the isolated WTAs could not^28^,^29^. Additionally, treating the cell wall preparations with either muramidase or amidase or using deacetylated cell walls destroyed the phage inactivation capacity of these preparations^30^,^31^. These observations suggested that peptidoglycan may participate in phage adsorption directly or indirectly by providing rigid support for WTAs. These data prompted us to re-examine phage adsorption with an extended set of *S. aureus* cell wall mutants. In particular, we aimed to investigate how phages interact with WTAs, and how peptidoglycan structures affect their adsorption.

**Figure 5:**
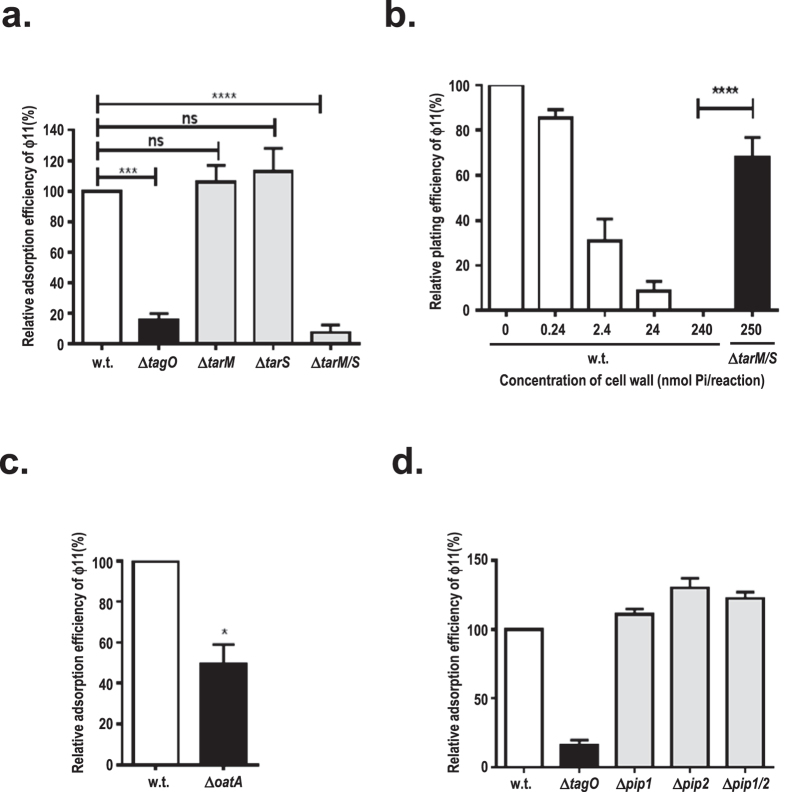
Efficiency of ϕ11 adsorption to *S. aureus* cell wall mutants. (**a**) Efficiency of ϕ11 adsorption to mutants with altered WTAs. The *S. aureus* wild-type strain (w.t., white bar) and mutants with altered WTAs were used as host. Mutants Δ*tagO*, Δ*tarM*, Δ*tarS*, and Δ*tarM/S* are indicated. (**b**) Dose-dependent inhibition of ϕ11 plating efficiency with wild-type cell wall but not the cell wall from mutant Δ*tarM/S* deficient in WTA GlcNAc residues. Wild-type cell wall concentrations used in this experiment range from 0 to 240 nmol Pi/reaction (white bars), and the concentration of mutant cell wall Δ*tarM/S* used in this experiment is 250 nmol Pi/reaction, which is indicated with black bar. (**c**) Efficiency of ϕ11 adsorption to Δ*oatA* mutant deficient in peptidoglycan acetylation. wild-type strain (w.t., white bar); Δ*oatA* mutant (black bar). (**d**) Efficiency of ϕ11 adsorption to *pip* mutants. wild-type strain (w.t., white bar), Δ*tagO* mutant (black bar), and *pip* mutants (grey bars) are indicated. Values are given as means ± standard deviations (SD, n = 3). Statistical significant differences calculated by one way ANOVA with Bonferroni’s post-test (Fig. 5a,b) or by the two-tailed Student’s paired t-test (Fig. 5c) are indicated: not significant (ns); *p < 0.05; ***p < 0.001; and ****p < 0.0001.

Adsorption assays were carried out using isogenic mutants with altered WTAs as hosts. As shown in Fig. 5a, ϕ11 virions were able to adsorb to either the Δ*tarM* mutant with only β-GlcNAc residues on WTA or the Δ*tarS* mutant with only α-GlcNAc residues on WTA with efficiency comparable to that of wild-type cells. In contrast, phage adsorption was significantly impaired when the Δ*tagO* mutant, devoid of WTAs, or the double mutant Δ*tarM/S,* deficient in both α- and β-GlcNAc residues on WTAs, were used as hosts (Fig. 5a). Consistent with these findings, the cell wall preparation from the wild-type strain dose-dependently inactivated phage, with full inactivation reached at a concentration of 240 nmol phosphate per reaction, whereas the cell wall preparation from double mutant Δ*tarM*/*S* exhibited significantly less inhibitory effect on plating efficiency at a similar concentration (Fig. 5b). Collectively, these observations demonstrate that GlcNAc residues on WTAs are essential for phage adsorption regardless of their anomeric configurations.

*S. aureus* cell wall preparations with deacetylated peptidoglycan fail to inactivate phage 52A^31^, which is also a serogroup B phage like ϕ11. To examine if peptidoglycan acetylation is involved in ϕ11 adsorption, the *oatA* mutant^32^ deficient in 6-O acetylation of muramic acid residues in peptidoglycan was used as a host for the adsorption assay. As shown in Fig. 5c, phage adsorption efficiency decreased to 50% when compared to the wild-type adsorption, suggesting that peptidoglycan acetylation favours ϕ11 adsorption.

### Pip homologues in *S. aureus* do not play a role in ϕ11 adsorption

Some phages require a membrane-embedded protein receptor for irreversible binding before the translocation of the phage genome into the host cell^8^. Previous studies on phage-resistant mutants derived from *L. lactis* identified the phage infection protein (Pip) as the membrane receptor for lactococcal phage c2^33^. YueB, the Pip homologue in *B. subtilis*, was also identified as the membrane receptor for siphophage SPP1^34^. Using the amino acid sequence of YueB or Pip as a probe, two homologues with conserved membrane topology and 40% similarity to YueB were identified from the *S. aureus* genome and designated as Pip1 (SAV2643) and Pip2 (SAV0283), respectively. To determine if these two membrane proteins are involved in ϕ11 adsorption, knockout mutants deficient in *pip1, pip2*, or both were generated. Interestingly, ϕ11 plates well on *pip* mutants, and no decrease in ϕ11 adsorption efficiency was observed when these mutants were used as a host (Fig. 5d), suggesting that Pip homologues in *S. aureus* are not involved in phage ϕ11 adsorption.

## Discussion

Research on *S. aureus* phages has a very long history that can be traced back to the early studies of bacteriophages. Since the discovery of bacteriophages, many *S. aureus* phages have been isolated, and these were classified into three families and a few major serogroups^2^. Before molecular techniques became available, *S. aureus* phages had been widely used for typing *S. aureus*. It was known for a long time that many *S. aureus* phages carry virulence genes and are required for *S. aureus* virulence and adaptation^2^. Despite comprehensive studies on phage genomes^15^ and the role of *S. aureus* phages in horizontal transfer of resistance and virulence genes among clones and species^12^, the molecular interactions mediating phage adsorption to the staphylococcal cell surface remain poorly understood.

The mechanism underlying *S. aureus* phage adsorption has often been assumed to be similar to that of phages infecting gram-negative bacteria. However, as gram-positive bacteria have a very different cell wall structure compared to that of gram-negative bacteria, phages infecting gram-positive bacteria may employ adsorption mechanisms different from those infecting gram-negative bacteria. Accounting for over 50% of the cell wall mass, WTAs are the most abundant surface molecules in the cell wall of bacteria belonging to the order *Bacillales,* which includes genera such as *Bacillus, Listeria* and *Staphylococcus*^7^,^35^. Hence, it is most likely that phages infecting bacteria of these genera need to interact with WTAs for successful adsorption.

In this study, we demonstrated that GlcNAc residues on WTAs are essential for ϕ11 adsorption regardless of their anomeric configurations. We also found that 6-O-acetylation of muramic acid residues in peptidoglycan is involved in ϕ11 adsorption. We showed that Gp45 and Gp54 are two baseplate proteins critical for ϕ11 infection, as both antisera can neutralize ϕ11 infection dose-dependently. Recombinant Gp45 inhibits ϕ11 adsorption in a dose-dependent manner and binds to glycosylated WTAs, demonstrating that Gp45 is the RBP of ϕ11. Unfortunately, recombinant Gp54 purified from *E. coli* was not stable and hence unsuitable for cell wall binding studies, and its functions could not be tested.

Staphylococcal pathogenicity islands (SaPIs) have an intimate relationship with temperate staphylococcal phages. Phages can induce the SaPI cycle, which allows the SaPIs to be efficiently encapsidated into special small phage heads commensurate with their size^10^. Previous mutational analyses of the genes present in the morphogenesis cluster of ϕ11 demonstrated that the Gp45 was essential for both the phage infectivity and transduction of its cognate SaPI^13^. Of note, Δ*gp54* seemed to lose its baseplates and failed to plate on *S. aureus*. Surprisingly, Δ*gp54* was still able to transduce SaPIs, although with a 100-fold reduction in transduction efficiency when compared with wild-type ϕ11. These results highlight that Gp45 is essential for the recognition process, while the presence of the Gp54 significantly increases the binding affinity between the phage and its receptor. As the N-terminus of Gp54 was predicted to be similar to that of BppU, which maintains the attachment of RBP to the baseplate core in TP901-1, it is tempting to speculate that Gp54 plays an important role in anchoring RBP in the baseplate.

Previously, a tail protein ORF636 from a serogroup A phage phiSLT was characterized as an adhesion protein that binds to poly-glycerolphosphate (GroP) chain of lipoteichoic acids (LTAs)^25^. Notably, ORF636 shares high homology with Gp45 (62% similarity) and the ORF636 sequence exists in all known serogroup A phages infecting *S. aureus*. However, it was shown that all tested serogroup A phages can still form plaques on a *S. aureus* mutant deficient in LTAs, but not on a mutant deficient in WTAs^11^, suggesting that WTAs but not LTAs are required for *S. aureus* phage infection. The tight binding of glycerol and glycerolphosphate for the RBPs suggested that LTAs could act as receptors for lactococcal phages^36^, however the structure of LTAs is well conserved and thought to be too simple to explain the different host specificities of various lactococcal phages. Recently, by mutational analysis, it was demonstrated that cell wall polysaccharide (CWPS) is the host cell surface receptor of tested lactococcus phages of different groups and that differences between the CWPS structures play a crucial role in determining phage host range^37^.

It is noteworthy that many phages need a protein receptor for adsorption, for example, Fuh A, OmpA, OmpC, LamB for *E. coli* phages, GamR, YueB for *Bacillus* phages and Pip for *Lactococcus* phage^38^. Interestingly, all these protein receptors are non-essential and many of them were identified by transposon mutagenesis. However, by screening a mutant library of *S. aureus* we were unable to isolate ϕ11-resistant mutants, which carry transposon insertions in genes encoding membrane proteins^39^. It is now generally acknowledged that carbohydrate recognizing phages possess a broad baseplate structure with multiple receptor binding sites. Conversely, phages with stubby ends or tail fibres, including the lactococcal c2 phages and the *Bacillus* phage SPP1, may recognize protein receptors on the cell surface^40^. The crystal structure of Gp45 was solved and it was found that Gp45 forms six trimers in the baseplates of ϕ11 and that each monomer of Gp45 contains a five-bladed propeller domain with a cavity that could accommodate a GlcNAc moiety (Koc *et al*., unpublished data). Hence, the presence of 18 receptor binding sites in the baseplate of ϕ11 suggests that its receptors are saccharides but not proteins.

Accounting for over 50% of the cell wall mass, WTAs are considered to be the most abundant surface molecules in *S. aureus* and have been implicated in various critical processes and interactions such as staphylococcal cell division, biofilm formation, β-lactam resistance, and staphylococcal pathogenesis^41^,^42^. Due to the in-homogeneity of WTA, its analysis has proven to be very challenging. Unlike research carried out on DNA, RNA or protein, methods available for studying WTA function are very limited. Despite technical limitations, a few WTA-interacting proteins such as FmtA^43^, WTA antibody, MBL^44^, and SREC-1^45^ have recently been identified. Here, we report Gp45 as a new WTA-interacting protein. Our results may eventually provide new tools for labelling and detecting the subdomain structures in the cell wall of *S. aureus.* Additionally, this study establishes a solid basis for the development of sensitive affinity-based infection diagnostics^46^ and therapeutics for MRSA infection.

## Materials and Methods

### Bacterial strains and growth conditions

*S. aureus* strains used in these studies are listed in Table 1. Bacteria were grown at 37 °C in BM broth (1% tryptone, 0.5% yeast extract, 0.5% NaCl, 0.1% K_2_HPO_4_, 0.1% glucose) under agitation.

### Construction of *S. aureus* mutants

The deletion mutants ∆*pip1,* ∆*pip2,* and ∆*pip1*/*2* were constructed by allelic exchange. For knockout plasmid construction, the primers listed in Table S1 in the Supplementary Information were used. For deletion of *pip1*, flanking regions were amplified with primer pairs pip1-F1-up/pip1-F1-dn and pip1-F2-up/pip1-F2-dn. Purified PCR products were digested with SalI/NheI and NheI/EcoRI respectively, and subsequently ligated into the SalI/EcoRI digested knockout vector pBASE6^47^. The resulting plasmid was used for allelic exchange^48^. For the construction of the *pip2* deletion mutant, a similar approach was pursued. The flanking regions of *pip2* were amplified with primer pairs pip2-F1-up/pip2-F1-dn and pip2-F2-up/pip2-F2-dn, digested with XbaI and ligated. Afterwards this marker-less knockout cassette was subcloned into pKOR-1, and the resulting plasmid was used for mutant construction via allelic exchange^48^.

### Overexpression and purification of the recombinant Gp45 and Gp54

Both *gp45* and *gp54* were amplified by PCR from *S. aureus* strain SA113, which is a ϕ11 lysogen. The primers used for the PCR reaction are listed in Table S1 in the Supplementary Information. The amplified *gp45* or *gp54* genes were subcloned into the expression vector pET28a between the NheI and XhoI sites. The resulting plasmids were transformed into *E. coli* BL21 for overexpression of Gp45 or Gp54. Both proteins were fused to a hexa-histidine-tag at the N-terminus to facilitate purification. After IPTG induction of the host cells, recombinant Gp45 was extracted and purified according to the procedure described previously^49^. Briefly, cells were lysed via ultrasonication (Digital Sonifier, Branson). After centrifugation at 38.000 × g for 55 min, cell debris was removed, and the supernatant containing recombinant Gp45 protein was loaded on a 5 mL Ni-NTA-column (GE Healthcare). Fractions containing Gp45 were pooled and concentrated to 1 mg/mL using Vivaspin 20 centrifugal concentrators with a molecular size cut-off of 50,000 (Sartorius, Göttingen, Germany). The concentrated sample was then loaded on a size-exclusion chromatography column SD200 pre-equilibrated with SEC-buffer containing 25 mM HEPES, 150 mM NaCl, 1 mM DTT. Fractions containing Gp45 were pooled and concentrated as pure Gp45 preparations. The purity and folding of the recombinant Gp45 were assessed with SDS-PAGE, Circular dichroism (CD) spectroscopy and dynamic light scattering (DLS). Gp54 was purified by the same procedure as for Gp45.

### Preparation of cell wall from *S. aureus* strains

The cell wall was extracted according to the procedure described previously^50^. Briefly, *S. aureus* overnight cultures were harvested by centrifugation at 5000 × g for 10 minutes. The cells were washed with 20 mM NH_4_Ac buffer (pH 4.8) and re-suspended in the same buffer. After disruption in a cell disrupter (Euler, Frankfurt am Main, Germany), the cell lysates were centrifuged at 5000 × g to remove the intact cells. The supernatant was collected as a crude extract of cell wall and mixed well with 5 mM MgSO_4_, 40 U/mL DNase and 80 U/mL RNase at final concentrations before overnight incubation at 37 °C. Next, to remove any cell membrane contamination, SDS was added to a final concentration of 2%, followed by ultra-sonication for 15 min. After heating at 65 °C for one hour, the cell wall preparations were washed six times with 20 mM NH_4_Ac buffer by centrifugation at 12,000 × g. Finally, the cell wall preparations were re-suspended in distilled water and quantified by measuring the amount of inorganic phosphate using the QuantiChromTM Phosphate Assay Kit (BioAssay Systems, USA) as described previously^39^.

### Bacteriophage experiments

Using the double layer soft agar method, ϕ11 was propagated with the indicator strain, *S. aureus* strain RN4220, as a host.

Phage plating efficiencies were determined to investigate the effects of Gp45, Gp54 anti-sera and cell wall preparations on the inactivation of ϕ11. In brief, 100 μL of ϕ11 (3 × 10^6 ^PFU/mL) was mixed with 100 μL of cell wall preparations or antisera of certain concentrations and incubated at 37 °C for 10 min. Samples pre-incubated without any cell wall preparations or sera served as controls. Next, the mixtures were diluted before plating on the indicator strain (*S. aureus* strain RN4220) using double agar overlay methods. After overnight incubation at 37 °C, the plaques were enumerated. The efficiency of plating was calculated relative to that of plating of ϕ11 pre-incubated without any sera or cell wall preparations.

Adsorption assays were performed according to the procedure described previously^11^. Briefly, 200 μL of *S. aureus* wild-type or mutant cells containing 8 × 10^7^ CFU were mixed with 100 μL of ϕ11 containing 3 × 10^5^ PFU and incubated at 37 °C for 15 min. The bound phages were separated from the free phages by centrifugation at 13,000 × g for 5 min. Adsorption was calculated by determining the number of PFU of the unbound phage in the supernatant and subtracting it from the total number of input PFU. Adsorption efficiency was expressed relative to the adsorption of wild-type strain RN4220. Each adsorption assay was repeated at least three times. To study the inhibition of adsorption by Gp45, cells were pre-incubated with the purified recombinant Gp45 of indicated concentrations for 15 min before adding phages to the host cells.

### Purification of ϕ11 and electron microscopy methods

Phage ϕ11 lysate was centrifuged at 73000 × g, 4 °C for two hours (Beckman Optima XL-80K). The resulting pellet was re-suspended in 500 μL of TMN buffer containing 10 mM Tris-HCl, pH 7.5, 10 mM MgSO_4_, 500 mM NaCl. The sample was then mixed well with 55% CsCl in TMN-buffer to give a final concentration of 42% CsCl and subjected to ultracentrifugation at 245,000 × g, 15 °C for 20 hours (Beckman). The visible phage band on the CsCl gradient was collected and sequentially dialyzed for two hours each in a D-Tube Dialyzer Mini (Novagen^®^, Merck Millipore, Darmstadt, Germany) against decreasing concentrations of NaCl in TMN buffer (10 mM Tris-HCl, pH 7.5, 10 mM MgCl_2_, 4 M NaCl) until the NaCl concentration after each round of dialysis was at 4 M, 2 M, 1 M and 10 mM NaCl, respectively.

For immunogold labelling, purified phage samples were adsorbed to glow discharged, pioloform and carbon-coated grids. The grids were then blocked with 0.2% gelatin in phosphate-buffered saline for 10 min followed by incubation with rabbit anti-Gp45 or rabbit anti-Gp54 serum, which were diluted in blocking buffer at 1:20 and 1:100, respectively. Polyclonal rabbit antisera were raised against purified recombinant Gp45 or Gp54 using a custom antibody service, Speedy 28-Day polyclonal program from Eurogentec (Brussels, Belgium). After blocking at room temperature for 60 min, the grids were washed six times with blocking buffer for a total time of 15 min before incubation with goat anti-rabbit IgG coupled with 12 nm gold colloids (Dianova, Hamburg), which was diluted with blocking buffer at 1:30. After incubation at room temperature for 60 min, the grids were washed three times with blocking buffer for 10 min and three times with phosphate-buffered saline for 10 min, followed by washing four times with double-distilled water for 2 min. Finally, the grids were negatively stained with 1% (w/v) aqueous uranyl acetate before examination with a JEM-1400Plus transmission electron microscope (JEOL, Japan)

### Flow cytometry analysis

Flow cytometry was carried out to evaluate the binding of recombinant Gp45 to the *S. aureus* cell surface. Purified recombinant Gp45 was labelled with biotin using the EZ-Link™ NHS-Biotin kit (Thermo Fisher Scientific). Biotin-labelled Gp45 was then incubated with *S. aureus* wild-type or mutant cells for 30 min with shaking at room temperature. Cells were washed and stained with strep-Alu488 (Invitrogen) for one hour at 4 °C. Finally, cells were fixed for flow cytometry analysis.

### Statistical analysis

Results are expressed as the means ± standard deviations from at least three independent experiments. Statistical analysis was performed using GraphPad Prism (GraphPad Software, Inc., La Jolla, USA, Version 5.04). Statistically significant differences were calculated with two-tailed Student’s t-test or one-way ANOVA with Bonferroni’s post-test as indicated.

## Additional Information

**How to cite this article**: Li, X. *et al*. An essential role for the baseplate protein Gp45 in phage adsorption to *Staphylococcus aureus. Sci. Rep.* **6**, 26455; doi: 10.1038/srep26455 (2016).

## Supplementary Material

Supplementary Information

## Figures and Tables

**Table 1:** Bacterial strains used in this study.

**Bacterial strain**	**Description**	**Reference**
BL21	*E. coli* BL21, host of inducible recombinant protein expression	Invitrogen
RN4220	*S. aureus* strain deficient in restriction, capsule, or prophage.	51
Δ*tagO*	RN4220, Δ*tagO*	11
Δ*tarM*	RN4220, Δ*tarM*	52
Δ*tarS*	RN4220, Δ*tarS*	52
Δ*tarM*/*S*	RN4220, Δ*tarM,* Δ*tarS*	52
Δ*pip1*	RN4220, Δ*pip1*	This study
Δ*pip2*	RN4220, Δ*pip2*	This study
Δ*pip1/2*	RN4220, Δ*pip1,* Δ*pip2*	This study
SA113	Derivative of *S. aureus* strain NCTC8325 harboring prophages ϕ11, ϕ12, and ϕ13	53
Δ*oatA*	SA113, Δ*oatA*	32
