# Supplementary material for: An essential role for the baseplate protein Gp45 in phage adsorption to Staphylococcus aureus
Source: Sci Rep. 2016 May 23;6:26455. doi: 10.1038/srep26455 (PMC4876445; doi:10.1038/srep26455)
Supplement: Supplementary Information [file srep26455-s1.pdf]

An essential role for the baseplate protein Gp45 in phage adsorption to *Staphylococcus aureus*

Supplementary information:

Xuehua Li[1], Cengiz Koç[2], Petra Kühner[1], York-Dieter Stierhof [3], Bernhard Krismer[1], Mark C Enright [4] , José R Penadés [5], Christiane Wolz [1], Thilo Stehle [2,6,8], Christian Cambillau[7], Andreas Peschel [1,8], Guoqing Xia [1,8,9,*]

1. Interfaculty Institute of Microbiology and Infection Medicine, University of Tübingen, 72076 Tübingen Germany,
2. Interfaculty Institute of Biochemistry, University of Tübingen, 72076 Tübingen, Germany
3. Center for Plant Molecular Biology , University of Tübingen, 72076 Tübingen, Germany
4. School of Healthcare Sciences, Manchester Metropolitan University, Chester Street, Manchester, M1 5GD, United Kingdom
5. Institute of Infection, Immunity and Inflammation, College of Medical, Veterinary and Life Sciences, University of Glasgow, Glasgow, United Kingdom
6. Vanderbilt University, School of Medicine, Nashville, TN 37232, USA
7. Architecture et Fonction des Macromolécules Biologiques, Centre National de la Recherche Scientifique, UMR 6098, Campus de Luminy, Case 932, 13288 Marseille Cedex 09, France
8. German Center for Infection Research (DZIF), partner site Tübingen, Germany
9. Institute of Inflammation & Repair, Faculty of Medical and Human Sciences, University of Manchester, Oxford Road, Manchester, M13 9PT, United Kingdom

∗ Correspondence to Dr. Guoqing Xia, e-mail: guoqing.xia@manchester.ac.uk

Supplementary Table S1: Primers used in this study.

Primer	Sequence
gp45-up (NheI)	CACG**GCTAGC**ATGAGTAATAAACTAATTACAGATTTAAGTAG
gp45-dn (XhoI)	CAGT**CTCGAG**TTATTCAACCACCTTTCCTTCGAAT
gp54-up	CACG**GCTAGC**ATGGTAGTAGATAATTTTTCGAAAGATGA
gp54-dn	CAGT**CTCGAG**TTATTTCCACCCCATAATTTTAGTTATAGT
pip1-F1-up	CTAGGATCTGTGTCGACTAATGATGTTG
pip1-F1-dn	GGAAGCTAGCTAGTCGATTGATACCTATC
pip1-F2-up	CCAAAGTAACGCTAGCGAATAAAAATT
pip1-F2-dn	CAGAATTCACATTTAAAAATACTTCAGCG
pip2-F1-up	GGGGACAAGTTTGTACAAAAAAGCAGGCTCGCGCCGGTGTCTTTATTC ACTTTG
pip2-F1-dn	GTCATCTAGAACTTTGGGATCCTTTTTCTGATTGATCTCC
pip2-F2-up	GTCATCTAGAGTATGTTCTTAATGATTGCTGCATTGGGTC
pip2-F2-dn	GGGGACCACTTTGTACAAGAAAGCTGGGTGAAACTGTCACGTAGTTCA GTTAATTCAGC

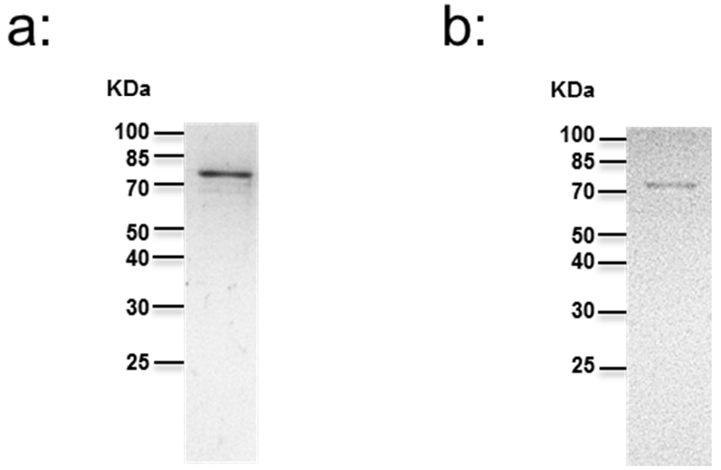

Supplementary Figure S1: Sodium dodecyl sulfate-polyacrylamide gel electrophoresis (SDS-PAGE) analysis of the purified Gp45 (**a**) and Gp54 (**b**) preparation from *E. coli*. The gel was stained with Coomassie brilliant blue R-250.
